# Accuracy and differential bias in copy number measurement of *CCL3L1 *in association studies with three auto-immune disorders

**DOI:** 10.1186/1471-2164-12-418

**Published:** 2011-08-18

**Authors:** Danielle Carpenter, Susan Walker, Natalie Prescott, Joost Schalkwijk, John AL Armour

**Affiliations:** 1 Centre for Genetics and Genomics and School of Biology, University of Nottingham, Nottingham NG7 2UH, UK; 2 Division of Genetics and Molecular Medicine, King's College London School of Medicine, 8th Floor Guy's Tower, Guy's Hospital, London SE1 9RT, UK; 3 Department of Dermatology, Radboud University Nijmegen Medical Centre, Nijmegen Centre for Molecular Life Sciences, 6525 GA Nijmegen, The Netherlands

## Abstract

**Background:**

Copy number variation (CNV) contributes to the variation observed between individuals and can influence human disease progression, but the accurate measurement of individual copy numbers is technically challenging. In the work presented here we describe a modification to a previously described paralogue ratio test (PRT) method for genotyping the *CCL3L1/CCL4L1 *copy variable region, which we use to ascertain *CCL3L1/CCL4L1 *copy number in 1581 European samples. As the products of *CCL3L1 *and *CCL4L1 *potentially play a role in autoimmunity we performed case control association studies with Crohn's disease, rheumatoid arthritis and psoriasis clinical cohorts.

**Results:**

We evaluate the PRT methodology used, paying particular attention to accuracy and precision, and highlight the problems of differential bias in copy number measurements. Our PRT methods for measuring copy number were of sufficient precision to detect very slight but systematic differential bias between results from case and control DNA samples in one study. We find no evidence for an association between *CCL3L1 *copy number and Crohn's disease, rheumatoid arthritis or psoriasis.

**Conclusions:**

Differential bias of this small magnitude, but applied systematically across large numbers of samples, would create a serious risk of false positive associations in copy number, if measured using methods of lower precision, or methods relying on single uncorroborated measurements. In this study the small differential bias detected by PRT in one sample set was resolved by a simple pre-treatment by restriction enzyme digestion.

## Background

Within the last few years there have been significant developments in our understanding of copy number variation (CNV) and its contribution to human genetic variation. It is now apparent that CNVs are both a frequent form of variation throughout the human genome, and are implicated in disease susceptibility [1,2].

In general, three forms of CNVs can be defined; insertion, deletion and multi-allelic, but accurate and standardised measurement of individual copy numbers is technically challenging. In particular the measurement of multi-allelic CNVs is the most problematic, more specifically those with higher copy numbers, where it is essential that the technical challenges of copy number genotyping are robust for all copy number classes and do not result in the systematic rejection of samples with high copy number. Repeated exclusion of particular genotypes will lead to mis-representation of copy number variation at a particular locus in a given population due to an artificially reduced apparent mean copy number. To define the full variation at a particular copy number variable locus it is critical that these technical challenges are overcome successfully.

Furthermore, and perhaps most importantly, accurate CNV measurement is essential in the context of case control association studies. Critically, inaccurate copy number measurements can lead to differential bias between cases and controls and result in false positive findings [3]. In SNP based studies differential bias can lead to differences in allele frequency estimates between batches of samples. In the context of CNVs bias can be seen as a systematic shift in the raw measurement around integer values for cases and controls, leading to differential mis-classification of samples by either over- or under-estimation of the integer copy number. Differences in DNA source and quality can cause small changes in the measured copy number distribution of case and control samples [2]. Furthermore, the power to detect disease associations in case control studies is also reduced with inaccurate genotyping of copy number [4]. Thus accurate determination of copy number is limited by the precision of the methodology used, which needs to be robust and replicable to give maximum power and reduce spurious disease associations.

New whole-genome genotyping platforms now incorporate probes to interrogate multiple CNVs [5], and have begun to yield associations between such variants and disease phenotypes [2]. However, even these methods are sensitive to DNA quality [6] and are limited in their accuracy to genotype multiallelic loci. The recent report by the Wellcome Trust Case Control Consortium [2] discusses in detail the potential artefactual influences on copy number determination and demonstrates the potential for reporting spurious associations with CNVs, especially multiallelic regions. To complement whole genome approaches, particularly for complex diseases where effect sizes are likely to be small, locus specific methods are also required for investigating multiallelic CNV loci. Such targeted methods can provide sufficiently accurate data to limit the potential for false positive or negative results as a result of differential bias. Locus specific methods are generally PCR-based, and perhaps the most widely used is Real-Time quantitative PCR, which depends upon the quantification of a copy variable test locus in comparison with an unrelated reference locus [7]. However, differential amplification efficiencies between test and reference loci can generate inaccuracies, particularly for high copy number measurements where the relative difference in ratio between test and reference products is smaller for neighbouring copy number classes. An alternative locus specific method with improved reliability for CNV determination is the paralogue ratio test (PRT) [8]. This method uses a single pairs of primers to amplify specifically two products simultaneously, one from a copy-constant reference locus and the other from the copy variable test locus of interest. The copy number of the test locus is then estimated from the ratio of test to reference products. Recently, the accuracy of the PRT method has been directly compared with a Real-Time quantitative PCR method of copy number measurement for β-defensins and shown to be the more accurate and robust approach [9]. The PRT method has previously been used to successfully analyse both β-defensins and *CCL3L1/CCL4L1 *copy numbers [10,11].

The copy variable genes *CCL3L1 *and *CCL4L1 *located on chromosome 17q12 lie in a 90 kb repeat unit, neighbouring the paralogous, but copy invariant, genes *CCL3 *and *CCL4 *[12]. *CCL3L1 *and *CCL4L1 *show 96% sequence similarity at both the nucleotide and protein level with their respective paralogues [13,14]. All four genes (*CCL3, CCL3L1, CCL4 *and *CCL4L1*) encode chemokines, chemotactic cytokines, which play an important role in the immune response by attracting lymphocytes and macrophages to sites of infection and inflammation. Furthermore they are all natural ligands for CCR5, the co-receptor used by HIV-1 for cell entry [15,16], with *CCL3L1 *being the most potent [17]. As such there have been a number of association studies focused on *CCL3L1 *copy number variation and HIV-1 susceptibility [7,18,19]. However, the reported associations are under dispute and the accuracy of *CCL3L1 *measurement is a major factor in the debate [20-23].

Here we report a modification of a previously described PRT method to measure *CCL3L1/CCL4L1 *copy number making it more efficient, cost effective and convenient. As *CCL3L1 *and *CCL4L1 *function as an attractant of inflammatory mediators we have performed three case control studies with autoimmune phenotypes (Crohn's disease, rheumatoid arthritis and psoriasis). We subsequently discuss in detail the accuracy of the PRT methodology used, the precision in *CCL3L1/CCL4L1 *copy number measurement and highlight the implications of differential bias with copy number variation.

## Results

### *CCL3L1 *copy number measurement

The paralogue ratio test was used to genotype the copy number of the *CCL3L1/CCL4L1 *copy variable region in a total of 1661 samples of European origin. The copy number measures generated with the "CCL3A" and "CCL3C" measurement systems were shown to be equivalent in accuracy (see additional file 1, Figure S1). In the majority of cases (1550/1661) the three independent PRT assays assigned concordant measurements of copy number for samples (to within 0.5 of the integer value for 85% of samples and to within 0.75 for 93% of samples) (see table 1). Samples showing a higher level of discordance between the integer copy numbers assigned by the PRT systems were assayed for two microsatellites that are present within the repeat unit [11]. The microsatellite genotyping allowed confident integer copy number calling for 31 of the discordant samples, leaving 2 samples for which the copy number cannot be confidently resolved, and these have therefore been excluded from further analysis.

**Table 1:** Breakdown of total numbers and concordance of PRT systems for the European samples by disease cohort

Disease cohort	No	Concordance (within 0.5)		Concordance (within 0.75)		Discordance	Missing data
Random UK controls	252	243	96%	252	100%	0	0

Crohn's disease	657	565	85%	607	92%	9 1.4%	41

Rheumatoid arthritis	274	189	69%	240	88%	12 4.4%	22

Psoriasis	478	408	85%	451	94%	12 2.5%	15

**TOTAL**	1661	1405	85%	1550	93%	33 1.9%	78

**Figure 1:**
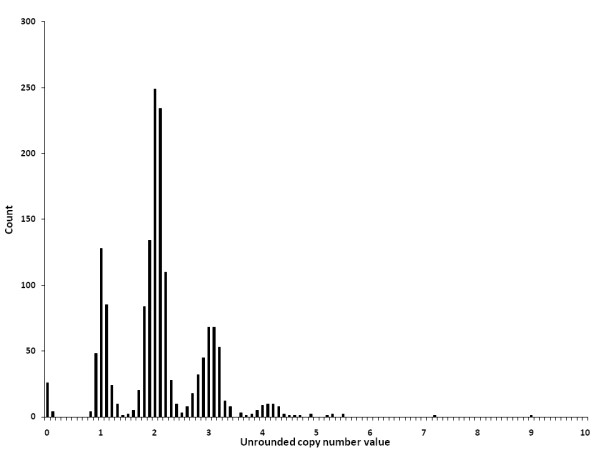
**Distribution of the unrounded copy number values for 1581 European samples**. The distribution shows a common range of 0-4 copies, with few high copy number outliers. The distribution shows peaks centred on the integer values with gaps between most clusters.

All 61 internal duplicate samples showed complete concordance of integer copy number, and an average calibrated copy number was generated from all PRT systems. For a total of 78 samples the PRT assays failed to produce sufficient signal, presumably due to low DNA concentration of these particular samples. The distribution of the unrounded copy numbers for 1581 samples is shown in Figure 1.

The different PRT systems (CCL3A/C, CCL4A and LTR61A) show a high level of agreement in all the European samples. This supports the previous observation by our lab [11] and suggests that discordancy between copy numbers of *CCL3L1 *and *CCL4L1 *as previously reported is rare [24]. Furthermore the agreement between all PRT systems indicates that variation in the copy number of the reference genes *CCL3 *and *CCL4 *is also uncommon in Europeans.

### Accuracy and error of PRT measurements

Germline copy numbers are integers, and thus we assume that precision and accuracy of measurement will be shown by a discrete distribution reflecting the underlying integer values [4]. The distributions of the raw data shown in Figure 1 clearly demonstrate PRT values clustering around the inferred integers. The overall standard deviation (normalised for copy number) of the full dataset was 0.075; and for each cohort separately the standard deviations were 0.069 for the European controls, 0.066 for Crohn's disease samples, 0.077 for rheumatoid arthritis samples and 0.081 for Dutch psoriasis and control samples. These values are both small and consistent between datasets, indicating that the variance inherent in the *CCL3L1/CCL4L1 *PRT measurement system is low, and the precision of the PRT measurements in estimating integer copy number is high and reproducible between datasets.

The mean and standard deviation, normalised standard deviations and predicted probability of error for the full dataset are shown in table 2. The data show consistently that the means for each copy number lie within 0.1 of the corresponding integer and that the standard deviations for each copy class specifically are sufficiently low that the probability of integer error is also small, further highlighting the reliability of the PRT system to accurately and reproducibly measure *CCL3L1/CCL4L1 *copy number. Though the estimates of the probability of error of copy number calling are small, there is an increase for higher copy numbers which is likely the result of smaller sample size and potentially the greater variance observed at the higher copy numbers.

**Table 2:** Mean, deviations and predicted probability of error for the unrounded copy number values for 1581 European samples

Copy Number	No	Mean	Standard deviation	Normalised Standard deviation	Specific deviation	Probability of error
0	30	-0.033	0.068			

1	300	1.033	0.097	0.096	0.102	7.2 × 10^-07^

2	877	2.021	0.142	0.071	0.101	4.9 × 10^-04^

3	313	3.012	0.178	0.059	0.103	4.9 × 10^-03^

4	49	4.071	0.203	0.051	0.108	1.9 × 10^-02^

5	9	4.977	0.346	0.069	0.155	0.149

6	1					

7	1					

8	0					

9	1					

### Differential bias

**Figure 2:**
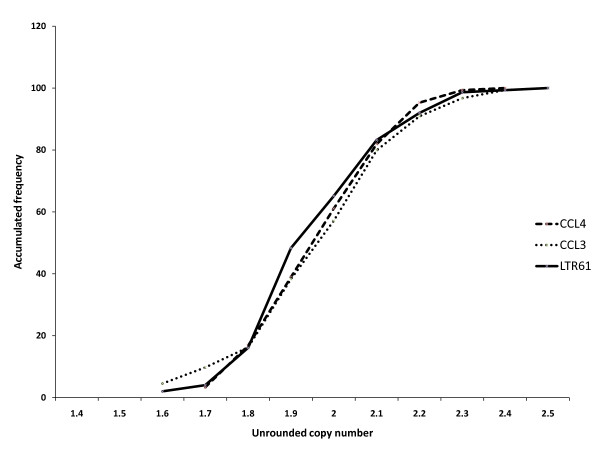
**A cumulative frequency plot of unrounded copy number values measured by the three PRT systems, (CCL3C, CCL4A and LTR61A) among samples assigned a copy number of 2 for the random UK control samples (149 samples)**. All three systems show a similar distribution around the mean value, displaying no obvious heterogeneity between the systems.

Differential bias in genotyping can lead to false positive or false negative associations in case control analyses and therefore it is essential that this is addressed in datasets. In particular, CNV measurements may be susceptible to shifts in results arising from physicochemical properties of DNA samples, properties which may be systematically different between cases and controls. We reasoned that if such shifts were present in our own data, they should be evident as different distributions of raw measurements around integer values. Examination of such distributions showed no significant difference between the three PRT systems (CCL3C, CCL4A and LTR61A) using the European control samples. A cumulative frequency plot of the unrounded copy number data for the predicted 2-copy samples is shown in Figure 2. This suggests that the raw data measurements from the three independent PRT systems perform equivalently and generate comparable measures of the copy number. Thus it is appropriate to compare the three systems and this will not lead to differential error in the scoring of the copy numbers.

**Figure 3:**
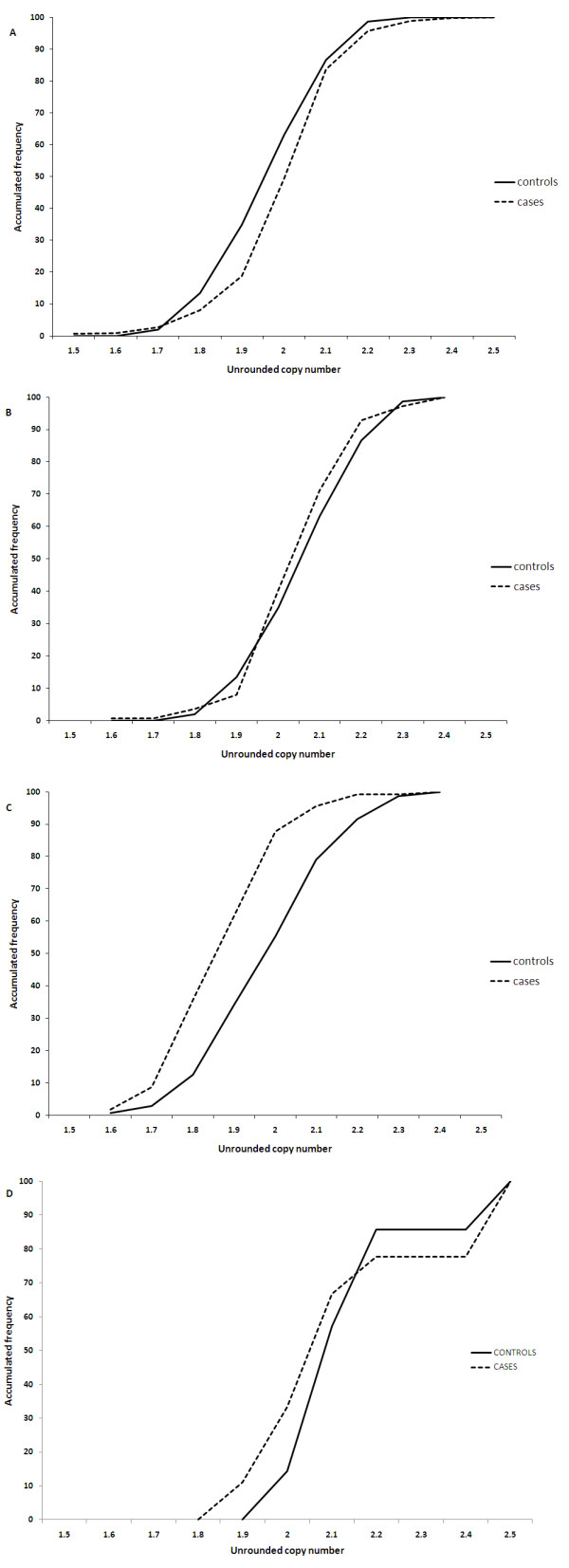
**A cumulative frequency plot for controls (solid black line) and cases (broken black line) for Crohn's disease samples (a), rheumatoid arthritis (b), and psoriasis (c) with copy number of 2**. There is no significant difference between the means of the distributions for the Crohn's disease and rheumatoid arthritis samples, whereas for the psoriasis samples the values for cases are significantly shifted towards lower values relative to the controls. Pre-digestion with the enzyme *BccI*, prior to PRT (d) shown here on a subset of 20 Dutch samples, resolves the significant difference between case and control samples.

Distributions around integer values were also compared between case and control samples for each clinical cohort. Owing to the precision of the PRT copy number measurement small, but systematic, differences in copy number measurement between cases and controls can be detected. Cumulative frequency plots were generated of the unrounded copy number distributions around the 2-copy integer for the clinical cohorts, shown in Figures 3a, b and 3c. The figures show a clear similarity between the case and control data for the Crohn's disease and rheumatoid arthritis cohorts. This suggests that there is no differential bias in these datasets and that the copy number genotyping is equivalent in the cases and controls. However, the cumulative frequency plot for the psoriasis cohort identifies a small but significantly different distribution of copy number measurement (p < 0.0001, t-test) between cases (mean = 1.91) and controls (mean = 2.03) (see Figure 3c). This difference between cases and controls was also observed for the 1-copy samples (p < 0.001) and 3-copy samples (p < 0.01) (data not shown).

### Basis of differential bias

Interestingly, two different DNA extraction methods were used for the psoriasis cases and controls. All the cases were extracted using a Qiagen kit, whereas the controls were extracted using a salting-out procedure. It has previously been suggested that different methods of DNA extraction between cases and controls can lead to differential bias due to differences in DNA quality and purity [6]. We envisaged that one potential physicochemical difference between genomic DNA preparations could be the level of residual protein, and in particular locally bound proteins that might be unusually tenacious in particular regions. We therefore tested whether separating our PRT amplicon from neighbouring DNA by restriction digestion might remedy the differential bias. Digestion with the restriction enzyme *Bcc*I leaves all PRT amplification sites used here intact, but separates them from neighbouring sequences that may potentially influence PCR efficiencies differentially. The discrepancy between cases and controls was rectified by pre-digestion of DNA samples with *Bcc*I: after digestion with *Bcc*I the cumulative frequency plot showed no significant difference between cases and control samples (data not shown), (two sets of control experiments were also performed; one set of samples was incubated at 37°C but lacked *BccI *and the other set of samples was incubated at 37°C with enzyme buffer present but not *BccI*. Differential bias was still observed with all these control samples). The differential bias did not alter the integer copy number calling of the samples. Although it may not be the cause of all or even most cases of differential bias, the observation that restriction digestion appears to mitigate differences between the case and control DNA collections does suggest that residual local protein binding might underlie the bias initially observed in this study.

### Association studies

Association analyses were performed for the different clinical cohorts. A two-tailed t-test was carried out to test for significant differences in the means between the Crohn's disease cases (n = 616), and European controls (n = 252); between the rheumatoid arthritis cases (n = 252), and European controls (n = 252); and between the psoriasis cases (n = 195), and the Dutch controls (n = 265), and no significant difference was found for all tests. The frequency of cases and controls with each copy number are shown in tables 3, 4, and 5 for Crohn's disease, rheumatoid arthritis and psoriasis respectively. The frequency distributions of cases and controls are shown in additional file 2 figure S2. These represent relatively small-scale association tests, and are only well powered to detect relatively large effects; for example, simulations show that the Crohn's disease cohort (616 cases and 252 controls) has about 90% power to detect an effect with an odds ratio of 1.5 at a significance level of 5%, and about 70% to demonstrate an effect at significance level 1%. By contrast, the same cohort would only have about 50% power to demonstrate an effect with significance level of 5% with an odds ratio of 1.3.

**Table 3:** Distribution of integer copy numbers in Crohn's disease cases and controls

Copy number	Cases	Controls
0	14 (2.27%)	4 (1.59%)

1	119 (19.34%)	49 (19.44%)

2	331 (53.73%)	149 (59.13%)

3	124 (20.13%)	45 (17.86%)

4	21 (3.41%)	5 (1.98%)

5	4 (0.65%)	

6	1 (0.16%)	

7	1 (0.16%)	

8	0	

9	1 (0.16%)	

TOTAL	616	252

**Table 4:** Distribution of integer copy numbers in rheumatoid arthritis cases and controls

Copy number	Cases	Controls
0	2 (0.79%)	4 (1.59%)

1	51 (20.24%)	49 (19.44%)

2	139 (55.16%)	149 (59.13%)

3	49 (19.44%)	45 (17.86%)

4	10 (3.97%)	5 (1.98%)

5	1 (0.39%)	

TOTAL	252	252

**Table 5:** Distribution of integer copy numbers in psoriasis cases and controls

Copy number	Cases	Controls
0	6 (3.08%)	4 (1.51%)

1	25 (12.82%)	56 (21.13%)

2	115 (58.97%)	143 (53.96%)

3	45 (23.08%)	49 (18.49%)

4	2 (1.03%)	11 (4.15%)

5	2 (1.03%)	2 (0.75%)

TOTAL	195	265

## Discussion

Over the last few years there has been a surge in interest in copy number variation in relation to human variation and disease and accurate copy number measurements are thus essential. Here we describe a more efficient modification of an already published method of locus specific copy number measurement for *CCL3L1/CCL4L1 *to ascertain copy number for a total of 1581 independent European samples.

We present here three association studies of *CCL3L1/CCL4L1 *measured with PRT, and find no association for variation in copy number with the autoimmune phenotypes Crohn's disease, rheumatoid arthritis or psoriasis. It is interesting to note however, that, for the Crohn's disease cohort, whilst the sample size is small, there are 7 cases with an integer copy number of 5 and greater, and yet there were no controls observed to have copy numbers greater than 4 (table 3). It has previously been reported that higher copy numbers of *CCL3L1/CCL4L1 *are associated with increased protein levels and enhanced chemotactic activity [24] which could suggest that higher copy numbers lead to increased recruitment of immune mediators and potential pathology. The role of both *CCL3L1 *and *CCL4L1 *predicts an influence of copy number on autoimmune disorders, but this was not supported by our data.

There is a previous report of an association between rheumatoid arthritis and *CCL3L1/CCL4L1 *copy number in a New Zealand cohort [25]. It is possible that the association study presented here for rheumatoid arthritis lacks power, but this association was also not supported by the recent report from the Wellcome Trust Case-Control Consortium (WTCCC) on CNV associations in eight common diseases [2].

Detailed analysis of the raw data generated from the PRT methodology shows the copy number genotyping to have a high degree of accuracy in integer copy number prediction, and that the level of accuracy is reproducible between European datasets. Inaccurate genotyping can lead to differential bias, a proportional shift in raw measurements that differ between sample batches, which we were able to investigate here due to the accuracy of the copy number measurement assay. Whilst evidence for differential bias was detected here with one of the case control cohort, this was resolved. This observation, however, does highlight the importance of examining raw data thoroughly prior to association analysis. Fortunately, the analysis presented here was with lower copy numbers and so the small differential bias observed did not alter the integer copy number calling. However it is clear that at higher copy numbers, or with a less precise measurement assay, differential bias could have easily caused frequent misclassification of integer copy number, potentially leading to false positive associations [3].

## Conclusion

In conclusion, the data presented here failed to find any association between disease and *CCL3L1 *copy number on three auto-immune disorders (Crohn's disease, rheumatoid arthritis and psoriasis) but did identify an example of differential bias. This was resolved by simple pre-treatment by restriction enzyme digestion; emphasising the importance of preparing case and control DNA together and encouraging the presentation of raw data to allow assessment of accuracy or bias in published association studies.

## Methods

### Samples

#### Control DNA samples

192 random control samples from the UK were used for direct comparison between the "CCL3A" and "CCL3C" PRT systems. These samples were from the European Collection of Cell Cultures (ECACC) Human Random Control (HRC) panels 1 and 2 (http://www.hpacultures.org.uk).

These ECACC samples were also used for the Crohn's disease and rheumatoid arthritis association studies, along with the parents (n = 60) from the International HapMap phase I CEPH samples (http://ccr.coriell.org), making a total of 252 random European control samples. All DNA provided was extracted from lymphoblastoid cell lines.

#### Samples from patients with Crohn's disease, rheumatoid arthritis and psoriasis

All samples were collected after informed consent and with appropriate ethical approval; details can be found in [9,10,26]. There were 657 Crohn's disease patient samples of UK origin, collected from patients attending clinics in London or Newcastle [9]. DNA was obtained from 274 rheumatoid arthritis patient samples of UK origin, although due to limited DNA volumes only 252 samples were successfully genotyped. The psoriasis samples and matched controls were from a cohort of samples from Nijmegen, Holland and have already been described [10]. In brief, there were 276 control samples from the Nijmegen Biomedical Study (NBS), and 202 unrelated psoriatic cases. All patients were diagnosed with psoriasis vulgaris, and classified as having moderate to severe psoriasis.

In order to address whether there is any population stratification of *CCL3L1 *copy number across Europe a t-test was performed between the Dutch control samples (n = 202) and the UK ECACC control samples (n = 192). There was no significant difference between the means of the two control datasets suggesting that that there is no population stratification across Europe and that the control samples are appropriate for the association studies.

### Copy number measurement

#### The paralogue ratio test (PRT)

*CCL3L1 *copy number of the psoriasis samples and the random control samples was measured using a minor modification of the PRT method previously described [11]. In the modified method the system "CCL3A" was changed to a similar system, termed "CCL3C", where the primer pairs for amplification of both *CCL3/CCL3L1 *were re-designed. The primers were specifically designed using DNA sequence flanking exon 1 of *CCL3 *and *CCL3L1*, and therefore will not amplify from the *CCL3L1 *pseudogene, which lacks exon 1. For the "CCL3C" PRT 1 μM each of primers FAM-labelled CCL3CF (GGC TAA GAC CCC TTC TAG AG) with CCL3CR (AAT CAT GCA GGT CTC CAC T) were used which gives products of 252 bp for *CCL3 *and 258 bp for *CCL3L1*. The "CCL3C" system PCR is more efficient than the "CCL3A" system and could be multiplexed with both the "CCL4A" and "LTR61A" systems in a single PCR reaction with a reaction mixture of 0.5 U Taq DNA polymerase (NEB) in a buffer with final concentrations of 50 mM Tris-HCl pH8.8, 12.5 mM ammonium sulphate, 1.4 mM magnesium chloride, 7.5 mM 2-mercaptoethanol, 125 μg/ml BSA and 200 μM each dNTP. PCR cycle conditions were 24 cycles of 95°C for 30s, 55°C for 30s, 70°C for 60s followed by a final hold at 70°C for 40 minutes. *CCL3L1 *copy number of Crohn's disease and rheumatoid arthritis samples were measured from genomic DNA using the paralogue ratio test (PRT) method previously described [11].

In order to reduce variation due to batch effects or plate position artefacts, case and control samples were interspersed within 96-well plates and genotyping was performed blind to the clinical status of each sample. Furthermore, all plates included some duplicate samples that were measured independently on separate plates to ensure consistency. For all PRTs fragment analysis of the test and reference loci was carried out by electrophoresis on an ABI3100 36 cm capillary using POP-4 polymer with an injection time of 30 s at 1 kV. Products from the PRT reactions were mixed with 10 μl HiDi formamide with ROX-500 marker (Applied Biosystems).

Two microsatellite PCRs were also performed, as described previously [11], for any sample showing inconsistencies between the PRT systems.

#### Copy number measurement of the PRT products

The peak areas were extracted using GeneMapper software (Applied Biosystems) and the ratio of test locus to reference locus was calculated for each sample. In order to calibrate and standardise unknown DNA samples, DNA samples of established copy number were included in each PCR in triplicate (ECACC samples, C0075 with a copy number (CN) = 1; C0150 with CN = 2; C0007 with CN = 3; and C0877 with CN = 4). A linear regression was applied to the cluster of ratios generated from the standard reference DNA samples and used to calibrate the inference of copy number from the ratios of the unknown samples. Copy number values from each of the independent PRT systems were compared and an arithmetic mean value was calculated from all PRT measurements to generate a single unrounded copy number value for each sample.

### Statistical analysis

#### Association studies

In order to test the difference in means between the copy number distributions of cases and control samples a two-tailed t-test was performed. Analyses were performed between European control samples and Crohn's disease or rheumatoid arthritis samples (both UK), and between Nijmegen psoriasis cases and Dutch controls.

#### Accuracy and error measurements

The accuracy of the PRT measurements to ascertain correctly the copy number for each sample was assessed in each dataset from the unrounded copy number data. All analyses were performed in Excel (Microsoft 2007) and SPSS (Version 16). For each dataset the mean and standard deviation of the unrounded data were measured for each copy number group. To obtain a general standard deviation of the whole dataset, and for each copy number class, the data were normalised by dividing the unrounded copy number value by the predicted integer copy number.

To estimate the probability of error inherent in a PRT system it is assumed that measurements of copy number values are normally distributed around each integer. From the observed data the mean and standard deviation for each copy number can be estimated and used to calculate the probability of mis-assigning a sample to the appropriate integer class.

To support the probability of misclassification approximation, a Kolmogorov-Smirnov test for normality was performed with the random control samples for copy number classes 1, 2 and 3. All 3 groups showed no significant deviation from normality (see additional file 3 figure S3 for Q-Q plots).

#### Differential bias

Differential bias was first examined for each PRT system (CCL3C, CCL4A and LTR61A) using the random control samples, to ensure that the different PRT systems are not differentially sensitive to DNA quality nor are there differences in PCR efficiencies that could lead to bias. Together this ensures that all the PRT data can be collated into a single value. Differential bias was also investigated between cases and control samples in the clinical cohorts, by comparing a normalised cumulative frequency plot of the unrounded copy numbers around an integer copy number of 2, as this is the most frequent copy number group, and therefore with greatest power to show any discrepancies. Where a difference was observed, a two-tailed t-test was performed to examine the significance of the deviation and other integer classes were investigated.

## Abbreviations

(CNV): Copy number variation; (SNP): single nucleotide polymorphism; (PRT): paralogue ratio test.

## Competing interests

The authors declare that they have no competing interests.

## Authors' contributions

DC undertook copy number genotyping and association analysis for the psoriasis samples, genotyping of the control samples, generated the differential bias data and drafted the manuscript. SW performed copy number genotyping and association analysis for Crohn's disease and rheumatoid samples, designed the new PRT measurement system CCL3C and revised the manuscript. JS provided the Dutch psoriasis case and control samples. JALA designed and coordinated the study, and revised the manuscript. All authors read and approved the final manuscript.

## Supplementary Material

Additional file 1 **Figure S1**. The distribution of unrounded copy number values for 192 ECACC samples typed with the "CCL3C" system, with discernable peaks around the integers, comparable to the original "CCL3A" distribution (figure 2a in Walker et al. 2009 [11]). The "CCL3A" method had an overall standard deviation of 0.087, whereas the modified "CCL3C" method had a standard deviation of 0.058 for the full dataset. Click here for file

Additional file 2 **Figure S2**. Histograms of the cases and control samples for Crohn's disease samples (a), rheumatoid arthritis (b), and psoriasis (c), with cases in black and controls in white. The histograms show no significant difference between the cases and controls for all datasets. Click here for file

Additional file 3 **Figure S3**. Q-Q plots of the control samples fitted to a normal distribution for copy numbers of 1 (n = 49) (control1) (a), 2 (n = 149) (control2) (b) and 3 (n = 45) (control3) (c). Click here for file
