# Supplementary figures and images for: Accuracy and differential bias in copy number measurement of CCL3L1 in association studies with three auto-immune disorders
Source: BMC Genomics. 2011 Aug 18;12:418. doi: 10.1186/1471-2164-12-418 (PMC3166952; doi:10.1186/1471-2164-12-418)

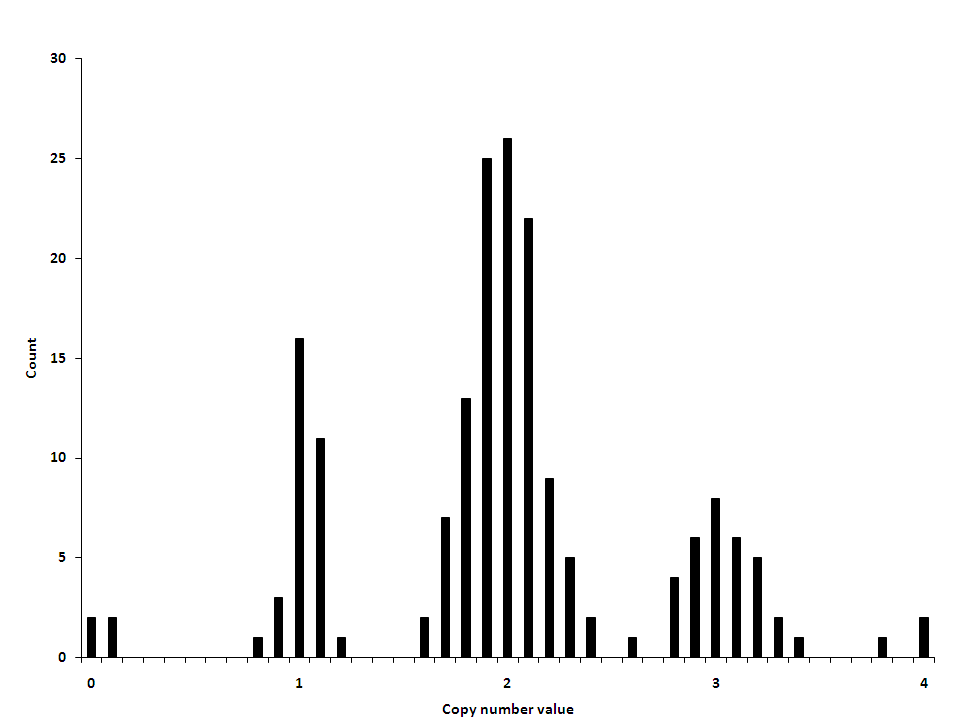

Supplement: Additional file 1 — Figure S1. The distribution of unrounded copy number values for 192 ECACC samples typed with the "CCL3C" system, with discernable peaks around the integers, comparable to the original "CCL3A" distribution (figure 2a in Walker et al. 2009 [11]). The "CCL3A" method had an overall standard deviation of 0.087, whereas the modified "CCL3C" method had a standard deviation of 0.058 for the full dataset. [file 1471-2164-12-418-S1.TIFF]

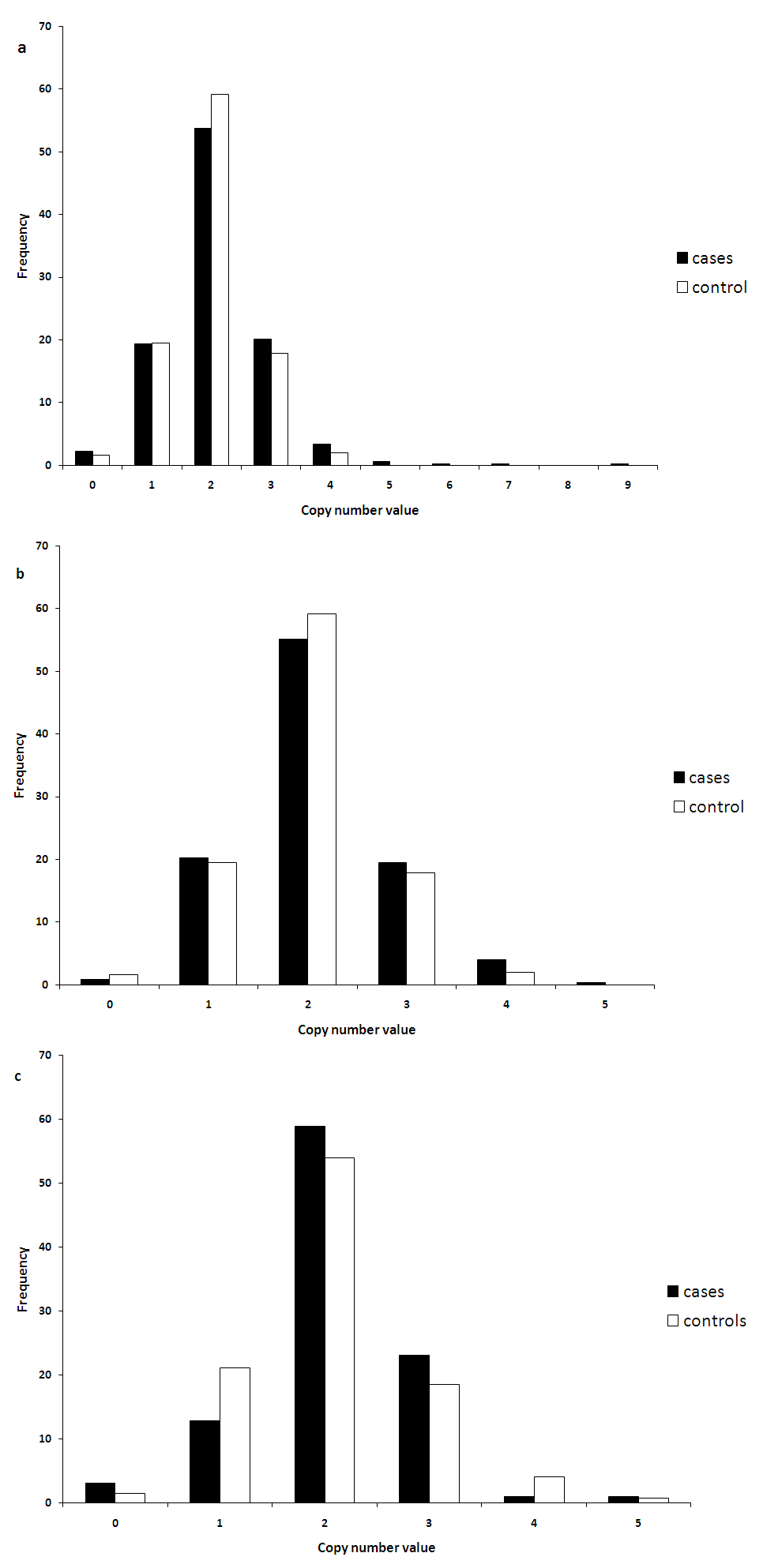

Supplement: Additional file 2 — Figure S2. Histograms of the cases and control samples for Crohn's disease samples (a), rheumatoid arthritis (b), and psoriasis (c), with cases in black and controls in white. The histograms show no significant difference between the cases and controls for all datasets. [file 1471-2164-12-418-S2.TIFF]

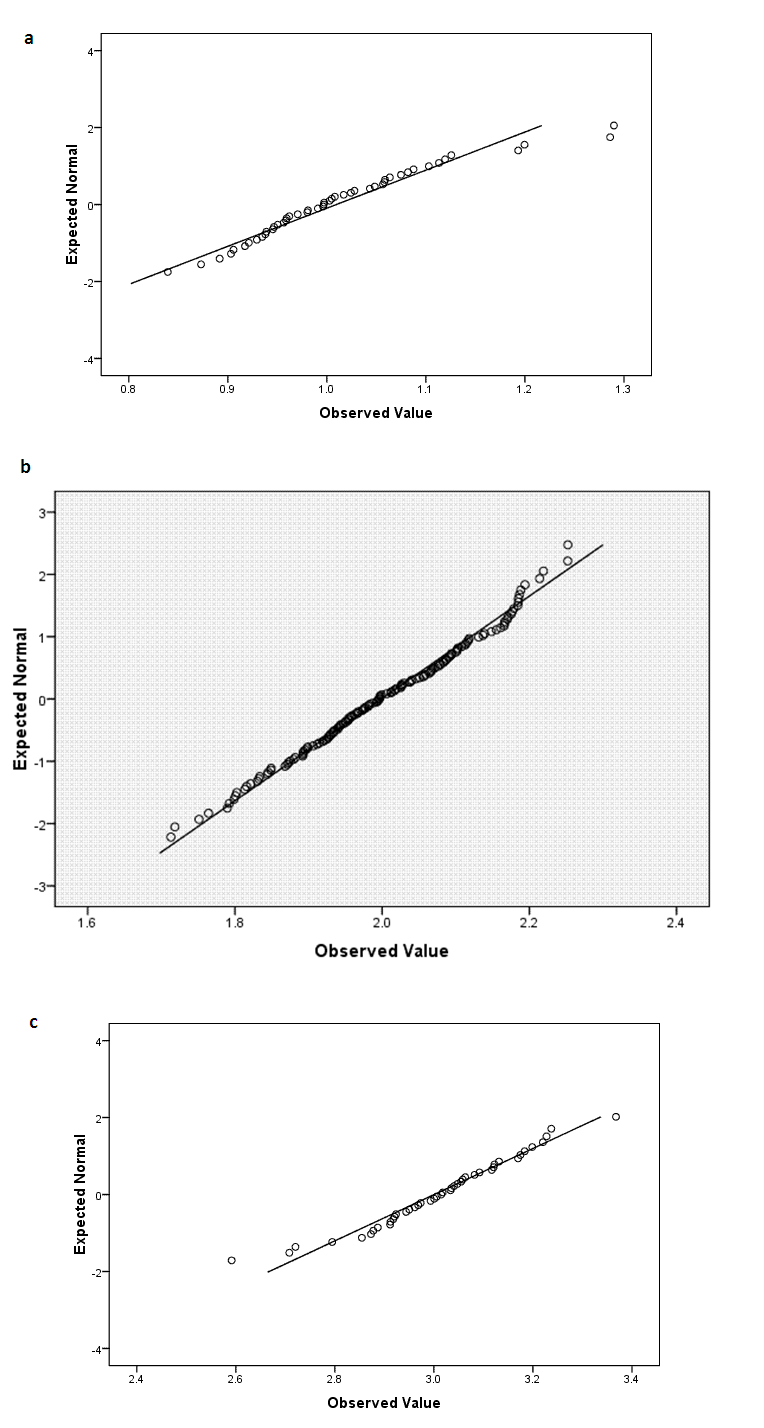

Supplement: Additional file 3 — Figure S3. Q-Q plots of the control samples fitted to a normal distribution for copy numbers of 1 (n = 49) (control1) (a), 2 (n = 149) (control2) (b) and 3 (n = 45) (control3) (c). [file 1471-2164-12-418-S3.TIFF]
